# Ocular TGF-*β*, Matrix Metalloproteinases, and TIMP-1 Increase with the Development and Progression of Diabetic Retinopathy in Type 2 Diabetes Mellitus

**DOI:** 10.1155/2021/9811361

**Published:** 2021-06-25

**Authors:** Lucia Saucedo, Isabel B. Pfister, Souska Zandi, Christin Gerhardt, Justus G. Garweg

**Affiliations:** ^1^Swiss Eye Institute, Rotkreuz and Retina Clinic, Berner Augenklinik am Lindenhofspital, 3012, Switzerland; ^2^Department of Ophthalmology, Inselspital, Bern University Hospital, University of Bern, 3012, Switzerland

## Abstract

Diabetic retinopathy (DR) is a sight-threatening late complication of diabetes mellitus (DM). Even though its pathophysiology has not been fully elucidated, several studies suggested a role for transforming growth factor- (TGF-) *β*, matrix metalloproteinases (MMPs), and tissue inhibitors of matrix metalloproteinase (TIMP) in the onset and progression of the disease. Consequently, the aim of this study was to analyze the concentrations of TGF-*β*1, TGF-*β*2, TGF-*β*3, MMP-3, MMP-9, and TIMP-1 in patients with different stages of DR in order to identify stage-specific changes in their concentrations during the progression of the disease. Serum and aqueous humor (AH) samples were collected during intraocular surgery, and eyes were classified into the following groups: healthy controls (*n* = 17), diabetic patients with non-apparent DR (*n* = 23), mild/moderate nonproliferative DR (NPDR) (*n* = 13), and advanced NPDR/proliferative DR (PDR) without vitreal hemorrhage (*n* = 14). None of the patients had been under anti-VEGF or laser treatment within six months prior to surgery. In the AH, TGF-*β*1 levels increased in advanced NPDR/PDR by a factor of 5.5 compared to the control group. Similarly, an increase in MMP-3 and TIMP-1 levels in the AH was evident in the later stages of DR, corresponding to a 7.7- and 2.4-fold increase compared to the control group, respectively, whereas serum levels of the studied proteins remained similar. In conclusion, increased concentrations of TGF-*β*1, MMP-3, and TIMP-1 in the AH, but not in the serum, in advanced NPDR/PDR indicate that the intraocular regulation for these cytokines is independent of the systemic one and suggest their involvement in the progression of DR.

## 1. Introduction

Diabetic retinopathy (DR) is one of the most common late complications of diabetes mellitus (DM) and the leading cause of vision loss among the working age population [1]. It is estimated that one in three people with DM has DR and that one in 10 will develop a sight-threatening form of the disease, including diabetic macular edema (DME) or proliferative DR (PDR) [2]. The most relevant factors associated with the prevalence and progression of DR are duration of diabetes, glycemic control, and hypertension [3, 4].

Transforming growth factor- (TGF-) *β* is a pleiotropic cytokine involved in cellular proliferation, differentiation, and migration, as well as in the production and degradation of extracellular matrix (ECM) components [5]. Three isoforms of TGF have been identified and are encoded by different genes but share 71−79% homology [6]. Even though the three isoforms of TGF-*β* have overlapping spatial and temporal expression patterns in several tissues, *in vivo* studies (as well as the isoform-specific knockout models) suggest that they may elaborate different tissue-specific functions [7, 8]. In the healthy retina, TGF-*β* is fundamental for the maintenance of vascular homeostasis, as it acts as a survival factor, inhibiting endothelial cell (EC) growth and migration and inducing differentiation and growth arrest in pericytes. Pathological TGF-*β* signaling has been suggested as one of the key mechanisms involved in the onset of DR [9, 10]. Dysregulation of TGF-*β* signaling leads to increased production of ECM components, which results in thickening of the basal membrane (BM) [11], one of the early hallmarks of DR. *In vitro*, TGF-*β* decreases the expression of VE-cadherin and claudin-5, leading to an increase in vascular permeability [12]. Moreover, inhibition of TGF-*β* signaling in experimental models is linked to vessel destabilization [13, 14].

In a similar way, matrix metalloproteinases (MMPs) have been linked to the pathophysiology of DR. MMPs constitute a multigene family of proteolytic zinc-dependent endopeptidases, which encompass at least 23 members in humans [15]. MMP activity is closely regulated by their endogenous inhibitors, tissue inhibitors of matrix metalloproteinase (TIMP), whose family encompasses four members. TIMP-1 is a strong inhibitor of most MMPs, including MMP-1, MMP-2, MMP-3, and MMP-9, and can, in addition, bind to pro-MMP-9, blocking the activation of the enzyme [16]. The balance between TIMP-1 and MMPs may be critical for tissue homeostasis in DR. Several studies have demonstrated the contribution of MMPs to the regulation of vascular permeability by degradation of junction proteins, namely, occluding [17, 18]. *In vitro*, the secretion of MMPs is induced by TGF-*β*, partially explaining the breakdown of the blood-retina barrier in advanced disease [19]. Latent TGF-*β*, in turn, is activated by MMP-2 and MMP-9. This indicates the complex interplay between TGF-*β* isoforms and MMPs in angiogenesis in general [20] and the progression of DR in particular.

Among the MMPs, MMP-2 and MMP-9 have been the most studied in the context of DR. Their involvement in the apoptosis of pericytes, ECs, and Müller cells [21, 22], as well as in the increase in vascular permeability [17, 23] and angiogenesis [24], has been demonstrated. To date, only limited information is available regarding MMP-3 in the retina in health and disease. The fact that it cleaves several ECM and BM components and is involved in the activation of other MMPs, namely, MMP-9 [25, 26], suggests a potential upstream regulatory role in DR. Interestingly, a pathological role of MMPs in several neurological diseases has been associated with its capability to degrade tight junction proteins and to compromise the blood-brain barrier [27, 28].

If TGF-*β*1, TGF-*β*2, TGF-*β*3, MMP-3, MMP-9, and TIMP-1 are involved in the pathogenesis of DR, as outlined above, we hypothesized that their intraocular and eventually also systemic concentrations might be linked to the severity of diabetes and DR. Therefore, this study is aimed at analyzing their concentrations in parallel samples of serum and aqueous humor (AH) from patients with untreated DR and healthy controls in order to identify stage-specific differences in their concentrations during the progression of DR.

## 2. Materials and Methods

### 2.1. Patients

This retrospective analysis refers to a total of 17 healthy individuals without any known systemic or ocular disease, except for requiring intraocular surgery (cataract, macular hole, or epiretinal membrane), and 50 patients with type 2 DM with or without DR undergoing intraocular surgery for the same reasons at a single institution (Clinic for Vitreoretinal Diseases, Berner Augenklinik am Lindenhofspital, Bern, Switzerland). Parallel samples of AH and serum were collected between 2013 and 2018 in a random fashion from healthy controls and diabetics irrespective of the severity and treatment of ocular and systemic diseases at the beginning of surgery. Any sample from patients meeting the inclusion criteria was included irrespective of the diabetic retinopathy stage if none of the following exclusion criteria were present: type 1 DM, history of any systemic malignant, vascular, or inflammatory comorbidity (e.g., rheumatic or autoimmune diseases), systemic treatments involving corticosteroids or immunomodulatory drugs, intravitreal or panretinal laser photocoagulation treatment within 6 months prior to surgery, vitreous hemorrhage, uveitis, glaucoma, or any unassociated concomitant retinal pathology.

The stage of DR was independently determined by a graduated ophthalmologist blinded to the study protocol based on the results of dilated stereobiomicroscopy of the anterior and posterior segments of the eye, macular optical coherence tomography (OCT), and widefield fundus images (Optos®) according to the International Clinical Diabetic Retinopathy Disease Severity Scale [29]. Ocular disease was correspondingly categorized as diabetes with non-apparent DR, mild/moderate non-proliferative DR (NPDR), and advanced DR (advanced NPDR/PDR).

This study was fully compliant with the tenets of the Declaration of Helsinki in its latest version and approved by the local Ethics Committee of the University of Bern (Ref. no 152/08). General informed consent was obtained from all study participants after the explanation of the nature and possible consequences of the study.

### 2.2. Determination of Target Protein Concentrations

After collection, serum and AH samples were immediately stored at -80°C until analysis. Samples were analyzed using a multiplex bead system, as previously described [30]. For the determination of target protein concentrations, the following assays were used: TGF-*β*1, TGF-*β*2, and TGF-*β*3 (Bio-Plex Pro TGF-*β* 3-Plex Assay, Bio-Rad, Hercules, CA, USA) and MMP-3, MMP-9, and TIMP-1 (Human Custom ProcartaPlex, Thermo Fisher, Waltham, Massachusetts, USA). The aforementioned TGF-*β* assay measures the active form of three TGF-*β* isoforms. The plates were read using the Bio-Plex FLEXMAP 3D system with xPONENT 4.2 software (Bio-Rad, Hercules, CA, USA). All procedures were performed following the manufacturer's instructions and in a blinded manner by an experienced technician.

### 2.3. Statistical Analysis

Measurements ranging below the lower limit of quantification (LLOQ) of the assay were replaced by half the value of the LLOQ specified for the corresponding target protein by the manufacturer, as previously established [31]. Outliers were identified by a box plot analysis (box whisker plot), and extreme outliers (more than three box lengths away from the edge) were excluded from the statistical analysis.

The Shapiro-Wilk test was applied to determine the normal distribution of the data. Since most data did not meet the criteria for normal distribution, the nonparametric Kruskal-Wallis test was used for intergroup comparison of continuous data and the chi-squared test of independence to evaluate variables measured at a nominal level. A *p* < 0.05 was considered to be significant. To control the risk of introducing type I error as a result of multiple comparisons, we applied the Holm correction, which progressively adapts the threshold for rejecting the null hypothesis. The statistical analyses were performed using the open-source software R (version 3.3.2 2016 RStudio, Inc.; psych package) and SPSS (version 23.0; IBM SPSS Statistics, Armonk, NY, USA). Results are expressed as the mean ± standard deviation (SD) (pg/ml) unless stated otherwise.

Since the majority of the target proteins in the AH were expected to range at the lower limit of the test system, we decided to use the absolute concentration values for statistical comparison, whereas the relative change of the targets between the three DR groups was compared to the healthy controls. These and the number of measurements ranging below the LLOQ were used for interpretation of their biological meaning.

## 3. Results

### 3.1. Demographic and Clinical Characteristics of the Study Population

Paired samples from a total of 67 eyes (67 patients) were included: healthy controls (*n* = 17), non-apparent DR (*n* = 23), mild/moderate NPDR (*n* = 13), and advanced NPDR/PDR (*n* = 14). The demographic characteristics of the patients are displayed in Table 1.

### 3.2. Serum and AH Concentrations of TGF-*β* Isoforms

**Figure 1:**
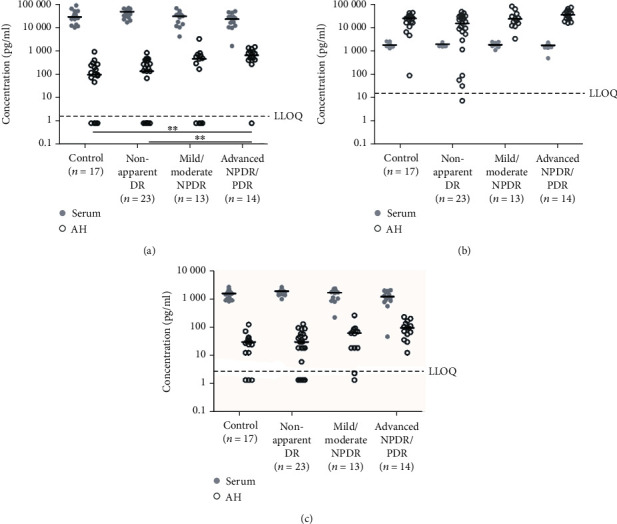
Scatter plots (log scale pg/ml) showing the concentrations of TGF-*β*1 (a), TGF-*β*2 (b), and TGF-*β*3 (c) in serum and AH throughout the different stages of DR. The values below the LLOQ were replaced with half the value of the LLOQ. Black lines represent the median value for each group. The dotted line shows the LLOQ of the assay for each protein. ^∗∗^*p* < 0.01.

The concentrations of TGF-*β* isoforms in the serum and AH throughout the different stages of DR are displayed in Table 2 and Figure 1. The mean ± SD concentrations and the relative changes for each group compared to the controls are presented in Table 2. Levels of TGF-*β*1 in serum were comparable between controls and all stages of retinal disease (Table 3, Figure 1), and no relevant differences in proportions were observed in the DR groups relative to the controls (Table 2). Patients with non-apparent DR showed higher levels of TGF-*β*1 compared to healthy controls and patients with advanced NPDR/PDR. However, these differences were no longer significant after the Holm correction. In the AH, on the other hand, we observed a stepwise increase of the TGF-*β*1 concentrations with increasing severity of DR compared to the controls (Table 2). There was a significant difference in concentrations between healthy controls and patients with advanced NPDR/PDR (where the relative change from healthy controls to patients with advanced NPDR/PDR was 5.5-fold). In addition, a significant difference between patients with non-apparent DR and advanced NPDR/PDR was found (Table 3).

Whereas all serum samples showed TGF-*β*1 concentrations above the LLOQ, these were generally low in the AH and ranged below the LLOQ in several samples, including the control group (4/17, 24%), non-apparent DR group (9/23, 39%), mild/moderate NPDR group (4/13, 31%), and advanced NPDR/PDR group (1/14, 7%). There was no difference between the groups regarding the number of concentrations below the LLOQ, neither for the AH nor for the serum in any of the TGF-*β* isoforms (chi-squared test of independence, *p* > 0.05).

Serum and AH TGF-*β*2 concentrations were comparable between the controls and the different DR severity groups (Table 3, Figure 1), with the exception of an increase in TGF-*β*2 levels in the AH between the non-apparent DR and advanced NPDR/PDR groups (*p* = 0.011), which did not remain significant after applying the Holm correction. No relevant differences in proportions were observed in the DR groups relative to the control group for TGF-*β*2 (Table 2). All serum samples showed concentrations above the LLOQ, and only one AH sample presented with concentrations below the LLOQ (non-apparent DR (1/23, 4%)).

Serum levels of TGF-*β*3 were comparable between the control group and the different DR groups. Similar to TGF-*β*1, the AH TGF-*β*3 levels increased with the progression of DR (Table 2), resulting in a 1.8-fold increase in the concentration of TGF-*β*3 in patients with mild/moderate NPDR and a 3.8-fold increase in patients with advanced NPDR/PDR compared to the controls. However, these differences did not remain significant after the Holm correction. No serum samples showed concentrations below the LLOQ. The number of values below the LLOQ in the AH for each group was as follows: control group (3/17, 18%), non-apparent DR (5/23, 22%), and mild/moderate NPDR (2/13, 15%) (chi-squared test of independence, *p* > 0.05).

### 3.3. Serum and AH Concentrations of MMP-3, MMP-9, and TIMP-1

**Figure 2:**
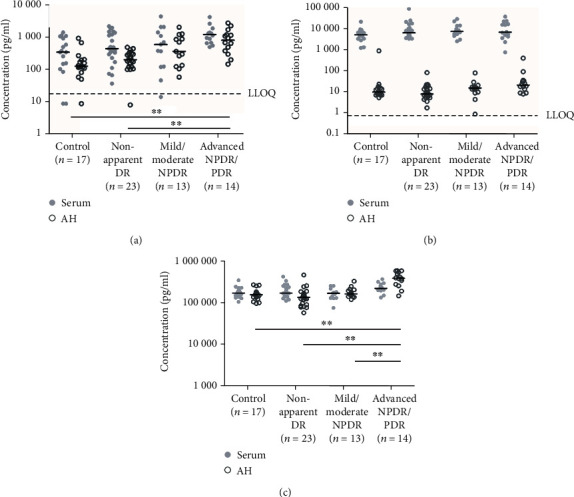
Scatter plots (log scale pg/ml) showing the concentrations of MMP-3 (a), MMP-9 (b), and TIMP-1 (c) in serum and AH throughout the different stages of DR. The values below the LLOQ were replaced with half the value of the LLOQ. Black lines represent the median value for each group. The dotted line shows the LLOQ of the assay for each protein. ^∗∗^*p* < 0.01.

The serum and AH concentrations of MMP-3, MMP-9, and TIMP-1 throughout the different stages of DR are presented in Table 4 and Figure 2.

The relative and absolute levels of MMP-3 tended to increase in the serum and AH with the advancement of DR compared to the controls (Table 4). In the serum, there was no significant difference in concentrations of MMP-3 between the groups. In the AH, we did, however, find a significant difference in the concentrations of MMP-3 between healthy controls and patients with advanced NPDR/PDR (where the relative change from healthy controls to patients with advanced NPDR/PDR was 7.7-fold), as well as a significant increase between the patients with non-apparent DR and advanced NPDR/PDR (Table 5).

All serum and AH concentrations ranged above the LLOQ, except for three values in the serum (control group (2/17, 12%) and mild/moderate NPDR (1/13, 8%)) and two values in the AH (control group (1/17, 6%) and non-apparent DR (1/23, 4%)). There was no difference between the groups regarding the number of concentrations below the LLOQ, neither for the AH nor for the serum in any of the MMPs or TIMP-1 (chi-squared test of independence, *p* > 0.05).

Serum concentration levels of MMP-9 were similar between the groups (Table 5), whereas the concentrations of MMP-9 in the AH tended to increase in the advanced stages of the disease, with a 2.3-fold increase in the advanced NPDR/PDR group relative to the control group. However, the difference between the control group and the advanced NPDR/PDR group did not remain significant after the Holm correction. No relevant differences in proportions were observed in the DR groups relative to the control group (Table 4). All serum and AH samples showed MMP-9 concentrations above the LLOQ. Again, there was no difference between the groups regarding the number of target concentrations above the LLOQ (chi-squared test of independence, *p* = 0.52).

Serum concentrations and proportions of TIMP-1 were similar in the healthy controls and the different DR stages (Tables 4 and 5, Figure 2), whereas a significant increase in TIMP-1 concentrations was observed in the later stages of DR (Table 5). All serum and AH samples showed detectable concentrations of TIMP-1.

## 4. Discussion

We were able to demonstrate an increase of TGF-*β*1, MMP-3, and TIMP-1 in the AH in the later stages of DR, whereas no changes in concentrations were found in the serum with the progression of DR. Thus, the development and progression of DR seems to comprise a local regulatory process within the eye that is independent of systemic disease. Our results are in alignment with previous studies showing that biological changes to the intraocular environment are already detectable before the first clinical signs of DR develop, and these increase with the severity of DR [32–34]. The stage-specific increase of these biomarkers, although not strong enough to support a central role of these cytokines for DR, confirms their involvement in the pathogenesis of the disease and may explain the observed basal membrane thickening [35]. These results might suggest a potential use of these proteins as biomarkers of diabetic retinopathy. However, their use as potential targets in drug therapy development needs to be evaluated using animal models.

Under physiological or healthy conditions, as in our control group, TGF-*β*2 is the most abundant and the only TGF-*β* isoform that is present in a higher concentration in the AH than in the serum, which is in line with previous reports [36]. Since we did not find changes in its concentration with the advancement of retinal disease, TGF-*β*2 likely plays a subordinate role in the pathogenesis of DR. This result, however, also highlights the importance of measuring TGF-*β* isoforms separately, since high concentrations of TGF-*β*2 may by far outweigh (even relevant) changes in the expression of the less abundant TGF-*β*1 and TGF-*β*3.

TGF-*β* is known to play a fundamental role in vascular quiescence, pericyte recruitment, and angiogenesis [9, 37, 38]. Serum concentrations of TGF-*β*1 in our series tended to be higher in diabetics without apparent DR compared to healthy controls, whereas lower concentrations were observed in the advanced NPDR/PDR stage. In line with these findings, higher serum TGF-*β*1 levels in patients with type 1 DM with NPDR compared to those without microvascular complications and healthy controls have been reported [39]. Conclusive clinical evidence, however, has not been established since different studies in type 1 DM reported heterogeneous results, with a tendency towards an increase in TGF-*β* serum levels with the duration of diabetes and its microvascular complications [40, 41].

While we found no difference in serum MMP-3, MMP-9, and TIMP-1 concentrations between the DR stages, we observed a pronounced upregulation of MMP-3 in the AH in mild/moderate NPDR and advanced NPDR/PDR, as well as an upregulation of TIMP-1 during the late stage of DR. These results are in line with previous studies that have reported elevated levels of TIMP-1 in VF in PDR [42, 43]. MMP-9 concentrations tended to increase in the advanced stages of the disease, although this significance was lost after the application of the Holm correction. Roles for both MMP-2 and MMP-9 in the later stages of DR have been expected [44, 45], and an increase in MMP-9 concentrations in the AH and VF was reported in the late stages of the disease [46, 47]. However, our results suggest that MMP-3 and TIMP-1 or the balance between MMP-9 and TIMP-1 may play a more predominant role already early in the pathogenesis of DR [48].

Studies in animal models demonstrated that MMP-3 is expressed in Müller cells in the retina of adult mice [49, 50] and predominantly in the ganglion cell layer (GCL) and inner nuclear layer (INL) neurons in the retina of adult rats [51, 52]. A pathological or beneficial role of MMP-3 in the retina has been described for several diseases based on animal models [50–53]. A local source for this enzyme fits well with the stronger correlation of AH compared to serum MMP-3 with advanced DR. However, whether MMP-3 has a causative or protective role in the pathogenesis of DR remains to be determined.

Whereas several studies compared AH and serum concentrations of different cytokines in DR and control groups [54, 55], only a few studies have attempted to correlate specific biomarkers to the severity of DR [56, 57]. Although currently there exists no clear concept as to the regulation of the retinal environment and the source of the local cytokinome, available evidence seems in favor of local rather than systemic factors driving the pathogenesis and progression of both DR and DME [55]. This would explain that the AH, but not serum, concentrations of TGF-*β*1, MMP-3, and TIMP-1 in our series changed with the severity of DR. However, brain-derived neurotrophic factor levels in the serum were reported to correlate more closely with the severity of DR than the corresponding AH levels [58], indicating that this cannot readily be generalized.

The strengths of this study include a well-defined DR severity in all cases, a relatively large sample size, the parallel workup of the serum and AH, and the exclusion of eyes with a history of recent vitreal hemorrhage, intravitreal treatment, or panretinal laser photocoagulation within 6 months of surgery. Its retrospective nature and the limited test system sensitivity may limit the understanding of cytokine concentrations in the context of the pathophysiological concept of this disease. Multiple bead assay systems generally lack the sensitivity of specific single-target ELISA. Therefore, the absolute concentrations of cytokines vary depending on the multiple bead assay system in use and cannot directly be compared between different studies. In an attempt to compensate for this limitation, we decided to not only report the absolute biomarker concentrations but also interpret the factor of change for each target between the different DR severity groups and the healthy controls. This approach could also provide an option to allow for comparison between different studies and differentiation between statistically significant and/or clinically relevant changes.

The demographic and clinical characteristics of the disease groups were similar and showed no differences, whereas the control group presented a different gender ratio. A statistical analysis for the studied proteins was carried out in the non-apparent diabetic retinopathy group, and no significant differences were observed between genders, indicating that the possibility of this bias to affect the outcomes and conclusions seems unlikely.

With the aim of identifying stage-specific differences in the concentrations of the targets in the early and late stages of the disease, the groups mild and moderate NPDR (early DR) and advanced NPDR and PDR (late DR) had to be merged in order to achieve sufficient statistical power for this analysis. The rigid case selection criteria applied here, on the other hand, may be a strength, outbalancing this grouping strategy. This, namely, affects the exclusion of patients in the advanced NPDR/PDR with vitreous hemorrhage and intravitreal treatment within the past 6 months, as anti-VEGF treatment can affect the levels of cytokines [59]. All except 3 patients (3/14, 21%) included in this group had received panretinal photocoagulation at some point, and only 4 patients (4/14, 29%) presented DME. An exploratory analysis revealed no differences in the studied targets between patients with and without panretinal photocoagulation and patients with and without DME (data not shown). This lack of difference is in agreement with a preclinical study which found no significant differences in TGF-*β*2 in the AH of rats after panretinal photocoagulation treatment [60].

## 5. Conclusion

In conclusion, we found that the concentrations of TGF-*β*1, MMP-3, and TIMP-1 in the aqueous humor, but not in the serum, are upregulated with the progression of DR, suggesting their contribution to the local regulation of DR.

## Figures and Tables

**Table 1:** Patient baseline characteristics.

	Healthy controls (*n* = 17)	Non-apparent DR (*n* = 23)	Mild/moderate NPDR (*n* = 13)	Advanced NPDR/PDR (*n* = 14)	*p* value
Age (years; mean ± SD)	64.6 ± 10.8	71.6 ± 9.6	72.1 ± 7.9	69.1 ± 9.5	0.14
Gender F (%)	16 (94.1)	11 (47.8)	6 (46.2)	7 (50)	0.01^∗^
Duration of diabetes (years; mean ± SD)	N/A	15.0 ± 12.9	14.0 ± 5.1	17.1 ± 10.4	0.50
Medication (*n*, %)					
Insulin	0	8 (34.8)	8 (61.5)	10 (71.4)	0.09
Metformin	0	15 (65.2)	7 (53.8)	7 (50)	0.62
Statin	0	16 (69.6)	8 (61.5)	6 (42.9)	0.32
Fibrate	0	1 (4.3)	1 (7.7)	0 (0)	0.59
Sartane	0	15 (65.2)	7 (53.8)	10 (71.4)	0.59

DR: diabetic retinopathy; *n*: sample size; PDR: proliferative diabetic retinopathy; SD: standard deviation; F: females. ^∗^Significant difference between healthy controls and all three of the DR groups.

**Table 2:** Concentrations (mean ± SD; pg/ml) of TGF-*β*1, TGF-*β*2, and TGF-*β*3 in serum and AH of healthy individuals (controls) and diabetic patients with different stages of DR. The fold change for each group was calculated relative to the control group.

	Healthy controls	Non-apparent DR		Mild/moderate NPDR		Advanced NPDR/PDR	
	Mean ± SD (pg/ml)	Mean ± SD (pg/ml)	Relative change^∗^	Mean ± SD (pg/ml)	Relative change^∗^	Mean ± SD (pg/ml)	Relative change^∗^
TGF-*β*1							
Serum	31,445 ± 16,231	46,825 ± 15,812	1.5	30,571 ± 18,709	1.0	25,267 ± 15,082	0.8
AH	137 ± 129	196 ± 230	1.4	361 ± 320	2.6	761 ± 450	5.5
TGF-*β*2							
Serum	1,747 ± 340	1,785 ± 243	1.0	1,687 ± 328	1.0	1,553 ± 404	0.9
AH	19,216 ± 11,105	14,844 ± 12,607	0.8	24,627 ± 19,266	1.3	32,398 ± 15,395	1.7
TGF-*β*3							
Serum	1,578 ± 536	1,882 ± 433	1.2	1,524 ± 650	1.0	1,273 ± 603	0.8
AH	27 ± 19	38 ± 36	1.4	49 ± 34	1.8	103 ± 66	3.8

^∗^Factor of change compared to healthy controls.

**Table 3:** Comparison of concentrations of TGF-*β*1, TGF-*β*2, and TGF-*β*3 in serum and AH of healthy individuals (controls) and diabetic patients with different stages of DR.

	1 vs. 2	1 vs. 3	1 vs. 4	2 vs. 3	2 vs. 4	3 vs. 4
TGF-*β*1						
Serum	*p* = 0.024			*p* = 0.027	*p* = 0.0019	
AH			**p** = 0.00036		**p** = 0.00036	
TGF-*β*2						
Serum						
AH					*p* = 0.011	
TGF-*β*3						
Serum					*p* = 0.023	
AH			*p* = 0.0019		*p* = 0.0033	

1: healthy controls; 2: non-apparent DR; 3: mild/moderate NPDR; 4: advanced NPDR/PDR. Significant results after the Holm correction are displayed in bold.

**Table 4:** Concentrations (mean ± SD; pg/ml) of MMP-3, MMP-9, and TIMP-1 in serum and AH of healthy individuals (controls) and diabetic patients with different stages of DR. The fold change for each group was calculated relative to the control group.

	Healthy controls	Non-apparent DR		Mild/moderate NPDR		Advanced NPDR/PDR	
	Mean ± SD (pg/ml)	Mean ± SD (pg/ml)	Relative change^∗^	Mean ± SD (pg/ml)	Relative change^∗^	Mean ± SD (pg/ml)	Relative change^∗^
MMP-3							
Serum	510 ± 457	762 ± 682	1.5	1,044 ± 1,254	2.0	1,187 ± 599	2.3
AH	131 ± 67	231 ± 123	1.8	647 ± 602	5.0	1,008 ± 791	7.7
MMP-9							
Serum	5,457 ± 2,518	9,137 ± 6,153	1.7	10,696 ± 8,062	2.0	12,266 ± 10,747	2.2
AH	12 ± 5	11 ± 7	0.9	15 ± 8	1.3	29 ± 24	2.3
TIMP-1							
Serum	188,378 ± 60,029	205,462 ± 78,693	1.1	176,173 ± 53,489	0.9	245,471 ± 66,851	1.3
AH	172,552 ± 55,975	141,695 ± 54,074	0.8	191,046 ± 59,229	1.1	415,028 ± 153,150	2.4

^∗^Factor of change compared to healthy controls.

**Table 5:** Comparison of concentrations of MMP-3, MMP-9, and TIMP-1 in serum and AH of healthy individuals (controls) and diabetic patients with different stages of DR.

	1 vs. 2	1 vs. 3	1 vs. 4	2 vs. 3	2 vs. 4	3 vs. 4
MMP-3						
Serum			0.013			
AH	*p* = 0.014	*p* = 0.011	**p** < 0.0001		**p** = 0.0004	
MMP-9						
Serum						
AH			*p* = 0.011		*p* = 0.0041	
TIMP-1						
Serum			*p* = 0.027			*p* = 0.027
AH			**p** < 0.0001	*p* = 0.014	**p** < 0.0001	**p** = 0.0002

1: healthy controls; 2: non-apparent DR; 3: mild/moderate NPDR; 4: advanced NPDR/PDR. Significant results after the Holm correction are displayed in bold.

## Data Availability

The data obtained in this study is available from the corresponding author upon request.
